# Isolation and culture of dental pulp stem cells from permanent and deciduous teeth

**DOI:** 10.12669/pjms.35.4.540

**Published:** 2019

**Authors:** Shagufta Naz, Farhan Raza Khan, Raheela Rahmat Zohra, Sahreena Salim Lakhundi, Mehwish Sagheer Khan, Nuruddin Mohammed, Tashfeen Ahmad

**Affiliations:** 1 Ms. Shagufta Naz, M.Sc., Department of Surgery, Department of Biotechnology, University of Karachi, Pakistan. Aga Khan University, Karachi, Pakistan; 2 Dr. Farhan Raza Khan, FCPS, Department of Surgery, Aga Khan University, Karachi, Pakistan; 3 Dr. Raheela Rahmat Zohra, Ph.D. Department of Biotechnology, University of Karachi, Pakistan; 4 Dr. Sahreena Lakhundi, Ph.D., Department of Surgery, Aga Khan University, Karachi, Pakistan; 5 Ms. Mehwish Sagheer Khan, M.Sc. M.Phil., Department of Surgery, Aga Khan University, Karachi, Pakistan; 6 Dr. Nuruddin Mohammed, PhD, FMFM, Department of Obstetrics and Gynecology, Aga Khan University, Karachi, Pakistan; 7 Dr. Tashfeen Ahmad, FCPS, Ph.D., Departments of Surgery and Biological & Biomedical Sciences, Aga Khan University, Karachi, Pakistan

**Keywords:** Dental pulp, Tissue explant, Mesenchymal stem cells, Permanent teeth, Primary / Deciduous teeth, Tissue engineering

## Abstract

**Objective:**

To isolate dental pulp mesenchymal stem cells (MSCs) from non-infected human permanent and deciduous teeth.

**Methods:**

It was an in-vitro experimental study. Human teeth were collected from 13 apparently healthy subjects including nine adults and four children. After decoronation dental pulps were extirpated from teeth and cultured via explant method in a stem cell defined media. Data was analyzed by descriptive statistics.

**Results:**

As above MSCs emerged exhibiting fibroblast-like morphology. In vitro culture was positive for 100% (9/9) and 75% (3/4) of the permanent and deciduous teeth respectively. First cell appeared from deciduous teeth pulp in 10±6.2 days while permanent teeth pulp took 12.4±3.7 days. Together, 26.6±3.6 and 24.5±3.5 days were required for permanent and deciduous tooth pulp stem cells to be ready for further assays.

**Conclusions:**

The protocol we developed is easy and consistent and can be used to generate reliable source of MScs for engineering of calcified and non-calcified tissue for regenerative medicine approaches.

## INTRODUCTION

Mesenchymal stem cells (MSCs) are attractive tools for tissue repair because of their differentiation capability and abundance in tissues.^1^ Virtually every tissue has tissue specific stem cells.^2^ Although, MSCs were first isolated in bone marrow,^3^ a decline is observed in using bone marrow MSCs while interestingly, an increasing trend is evident regarding exploration of various postnatal tissues as a source of MSCs.^4^ Growing evidence suggests a remarkable regenerative potential of MSCs from dental derived tissue such as Dental pulp stem cells (DPSCs) from impacted third molar,^5^ stem cells from apical papilla (SCAP)^6^ and stem cells from human exfoliated deciduous teeth (SHED).^7^

Permanent and deciduous (primary) teeth are considered as an easily accessible source for isolation and subsequent expansion of dental pulp cells for the purposes of tissue engineering. The collection of dental pulp is an easier and safer undertaking than the collection from bone marrow.^8^

Since the discovery of dental pulp stem cells in 2000, these have been investigated extensively through *in-vitro* and *in-vivo* approaches. Very recently, researchers in Japan have conducted a phase I clinical trial to evaluate the safety of autologous dental pulp stem cells for regenerating dental pulp (DP).^9^

In Pakistan, the potential of dental pulp stem cells has yet to be explored in pre-clinical and clinical trials. Therefore, in the present study, we aim to develop a protocol for the isolation and culture of dental pulp stem cells from permanent and deciduous teeth with a long term goal of their use in regenerative medicine applications.

## METHODS

### Recruitment and Collection of Teeth Samples

This was an in vitro study on human samples. Patients were recruited from dental clinics at Aga Khan University Hospital, Karachi. Inclusion criteria for permanent teeth selection were healthy adult subjects; aged between 18 to 85 years, presenting for extraction of un-diseased third molars or orthodontic patients needing extraction of sound premolars for braces treatment. The inclusion criteria for deciduous teeth were healthy children; aged between 9 to 12 years, presenting with teeth near to physiologic tooth exfoliation. Only those deciduous teeth were selected whose dental pulp was sound and without any carious exposure. Written informed consent was obtained from adult subjects and assents from parents of children in English, or their native language. The study was approved by institutional Ethics Review Committee (ERC), Ref number 4-1997-BBS-ERC-12. The exodontia of teeth was performed under local anesthesia in all subjects except two. One adult subject underwent tooth extraction under general anesthesia and one child subject shed off tooth by gentle manipulation without any need for local anesthesia.

### Sample Processing

**Fig. 1:**
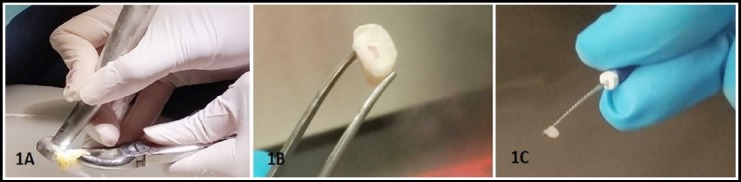
Collection of dental pulp tissue from extracted tooth. (1A) Cutting procedure of extracted tooth after disinfection; with the help of a stable finger support using dental fissure burs, the tooth was decoronated till cemento-enamel junction. Decoronation is an exothermic process therefore, an ample amount of sterile water was sprayed to reduce the heat. (1B) Removal of crown exposed the pulp chamber. (1C) Extirpated dental pulp from adult tooth on the tip of dental file.

A total of nine (n=9) permanent and four (n=4) deciduous teeth were collected. After disinfecting with 3% sodium hypochlorite solution for two minutes, tooth was rinsed with 1X phosphate buffer saline (PBS) and dried using cotton gauze. A cut around the cemento-enamel junction was made using a sterilized dental diamond fissure burs (Mani, Inc. USA) along with high speed hand piece (NSK, USA) under copious water supply to decoronate the tooth to expose the pulp chamber as shown in Fig.1A & 1B.

**Fig. 2:**
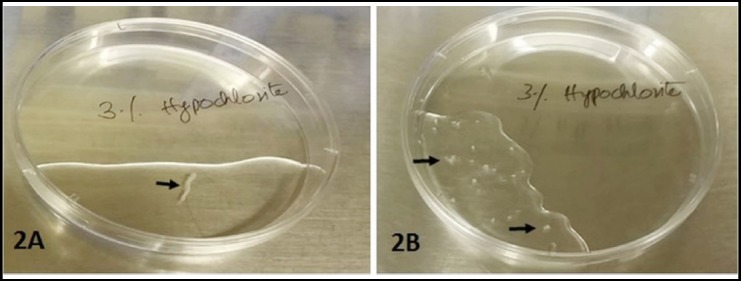
Dental pulp tissue in 1X PBS before (2A) and after (2B) mechanical mincing (arrows).

Sectioned teeth were placed into the transport media (TM) containing basic medium Dulbecco modified essential medium F12 (DMEM-F12) supplemented with 20% fetal bovine serum (FBS) and penicillin 500U/mL, streptomycin 500µg/mL, amphotericin B 1.25µg/mL) (Sigma Aldrich, Merck, USA). Samples then placed on ice were transferred to Juma Research Laboratories at Aga Khan University Hospital for subsequent processing and culture. Using aseptic condition, 100 mm petri plate (Sterilin) were set up for processing of each tooth in a biohazard laminar flow hood. Extracted tooth was decanted in a petri plate. Tooth was hold with the help of a sterile forceps and gently extirpated out DP tissue using endodontic H-file # 30 (MANI, Inc, USA). DP tissue was placed in 1X PBS containing 1% antibiotic antimycotic solution (Sigma Aldrich) in a petri plate for 10 to 20 minutes as seen in Fig. 2A and was washed twice with 1X PBS (Sigma Aldrich, Merck, USA) each for 10 minutes. Then were transferred into a new petri plate containing DMEM-F12 with 20% FBS. Minced into 1- 2mm^3^ pieces using surgical blade # 20 (Feather, WAPI, USA) as demonstrated in Fig.2B. DP minced fragments were plated in a T-25 flask (Thermo Scientific, USA) containing DMEM-F12 supplemented with 20% FBS, penicillin 100U/mL, streptomycin 100µg/mL, amphotericin B 0.25µg/mL, 1mM sodium pyruvate and 2mM L-glutamine (Sigma Aldrich). Explants were cultured at 37°C in a humidified incubator with 5% CO_2_. Cultures were observed daily under inverted microscope (Olympus Corp, USA) for any contamination and cell growth via migration from explant. Micrographs were captured using DSL3 standalone microscope camera controller (Nikon, Japan) at different magnifications.

### Passage

When cells reached 70 to 80% confluency they were either used for an assay or cryopreserved for later use. Cells were thoroughly washed with 1X PBS twice, tryspinized with 0.05% Trypsin-EDTA (Sigma Aldrich) for two to five minutes, neutralized by adding 10% FBS containing DMEM-F12. Detached cells were transferred in a tube, centrifuged at 500g for five minutes. Carefully decanted the supernatant, make sure not to dislodge the cell pellet. Cells were resuspended either in growth media for an intended assay or stored using freezing media containing 90% FBS and 10% DMSO (Sigma Aldrich) in liquid nitrogen at -196°C for later use or long term storage.

### Viability Assay

Cells were grown till reached 60-65% confluency. Briefly, cells were tryspinized and resuspended in complete media. Equal volumes of cell suspension and 0.4% trypan blue (Gibco) were mixed, 10µL of prepared sample were loaded in both chamber of hemocytometer. Viable and non-viable cells were counted within five minutes of preparing sample.^10^ Counting was performed in duplicate.

### Statistical Analysis

Data was analyzed using mean ± SD.

## RESULTS

**Fig. 3:**
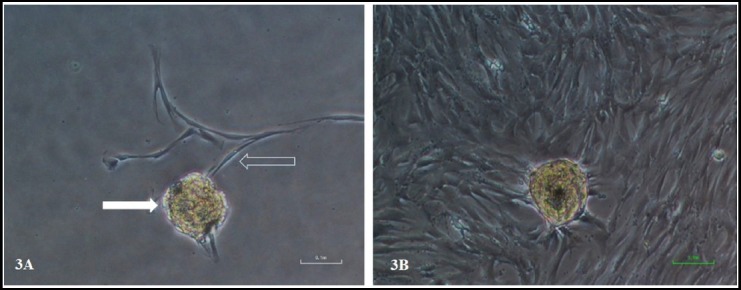
Dental pulp stem cells emerging from tissue explant. (3A) The micrograph showed the dental pulp tissue explant (solid arrow) and newly emerging cells from tissue explant (hollow arrow) at day 11 of culture. The cells exhibit typical morphology of fibroblast like cells with long cytoplasmic extension. (3B) showed same tissue explant after 19 days of culture with confluent field of view. Magnification 100X.

DP tissues were extirpated from tooth by decoronation as shown in Fig.1C and were cultured via outgrowth / tissue explant method. In terms of cellular morphology, cells migrated out of the tissue explant, exhibited homogenous morphology having a typical fibroblast-like shape, with long cytoplasmic processes as shown in Fig.3A & 3B.

The in vitro explant culture was positive for 100% (9/9) and 75% (3/4) permanent and deciduous teeth respectively. One pulp tissue from deciduous teeth didn’t show cell grow at all. The reason might be the osmotic shock to the pulp tissue due to sample transportation without transport media. The other pulp tissue grew into small colonies however, got contaminated during long primary culture. Both cell type exhibited low proliferative potential. DPSCs and SHED slightly differ in their growth rate. Deciduous teeth pulp gave rise first cell in 10 ± 6.2 days of culture while pulp from permanent teeth took 12.4 ± 3.7 days for the emergence of first cells. Together, 26.6 ± 3.6 and 24.5 ± 3.5 days were required for DPSC and SHED to be ready for further assays Table-I. Two pulp tissues, derived from a permanent and a deciduous tooth, were subjected to delayed processing of 21 and 24 hours respectively. In contrast to an average of 10 ± 6.2 days, the first cell appeared from deciduous teeth pulp tissue was increased to 17 days. However, the change due to delay in processing was not significant in case of pulp from permanent teeth.

**Table I:** Tabular summary of isolation and culture of dental pulp stem cells derived from permanent and deciduous teeth.

Type of Teeth	In-vitro Culture	First Cell Appearance (days)	Sub-culture (days)	Primary culture Duration (days)
Permanent (n=9)	Positive (100%)	12.4 ± 3.7	13.9 ± 4.2	26.6 ± 3.6
Primary (n=4)	Positive (75%)	10 ± 6.2	13.5 ± 4.9	24.5 ± 3.5

**Fig. 4:**
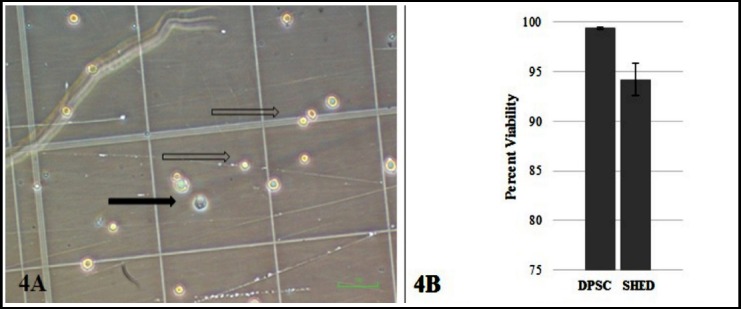
Trypan blue dye exclusion test showed that dental pulp MSCs remain viable after cryopreservation. (4A) hemocytometer counting chamber viable (hollow arrow) and non viable cell (solid arrow). (4B) percent viability of DPSC and SHED after cryopreservation. Error bar indicates standard deviation.

Cellular viability of DPSC and SHED was observed using trypan blue dye exclusion test indicating the cell membrane integrity was preserved during cryopreservation as shown in Fig.4. We found 99.4±.09% and 94.19±1.65% viability for DPSC and SHED respectively.

## DISCUSSION

We report a protocol that describes the isolation and culture of human dental pulp mesenchymal stem cells from permanent and deciduous teeth using explant method. DPSCs obtained from enzymatic and explant methods were reported to be comparable in terms of mesenchymal stem cell marker expression and multi-lineage differentiation thus suggested to be used as suitable autologous source to regenerate bone and cartilage.^11^

The emerging cells from tissue explant form loose colonies, comprised of cells with characteristic spindle shaped morphology, unlike epithelial cells which forms compact colonies. Our data are consistent with the results of other studies in which a homogenous population of cells were observed using tissue explant method.^12^,^13^ Interestingly, the protocol we developed is equally useful for teeth samples stored for up to 24 hours at 4°C. This is especially important, where a delay in sample processing is unavoidable. We presume such a strategy may be employed in setting up a tooth bank as reported elsewhere.^14^

The explant method has been extensively used for studying the dental pulp cells physiology, cell subpopulations capable of differentiating into odontoblasts or mineral-forming cells *in vitro^15^* and dentin-like structure *in vitro*.^16^ DPSCs and SHED obtained from enzyme digestion were also capable of differentiating into odontoblast-like cells and produced dentin *in vivo*.^6^,^7^ Furthermore, an immature DPSC (iDPSC) population was obtained from SHED via explant method. These cells expressed embryonic stem cell markers and showed dense engraftment.^17^ Although both methods yield similar cell populations, probably, the advantage of employing enzyme is that it releases all different types of cells present in the DP tissue.^15^

Primary culture of DPSCs grows slowly,^15^ our cells also took longer time to grow in culture. We also performed enzymatic digestion by treating minced DP tissue with 3 mg/mL collagenase type 1and dispase type II. However, it gave low cell yield which remained unable to grow. In our experiments, the undigested tissue fragments seemed to be the sole source of cells in culture (data not shown). Further, the use of undigested tissue to obtain dental stem cells has also been mentioned in literature.^18^ Cryopreservation protocol if not followed properly may result in reduced viability of stored cells. The protocol we used was effective for maintaining more than 90% viability of dental MSCs.

Extracted teeth, considered as clinical waste, offer a promising source of autologous cells. These cells can potentially be used for regeneration of musculoskeletal^19^ chondrocytes, adipocytes, cornea, hair follicle, and endothelial cells.^20^,^21^ Previous studies demonstrated the transdifferentiation of human dental pulp cells into neuron-like cells,^22^ oligoprogenitors,^23^ cardiomyocytes,^24^ and insulin producing cells.^25^ Above mentioned studies provide evidence in favor of regenerative potential of dental pulp MSCs.

Considering the most accessible and feasible cell source, the usefulness of dental pulp stem cells or dental cells in generating iPSCs has also been documented in literature. SHED, SCAP,^26^ and DPSCS can easily be reprogrammed into iPSCs at relatively higher rates.^27^ Very recently, human dental stem cell derived transgene-free iPSCs were successfully used to generate functional neurons, exhibiting sodium and potassium currents, action potential, or spontaneous excitatory postsynaptic potential.^18^

We report a protocol for the extirpation of dental pulp tissue, *in-vitro* explant culture and propagation of dental pulp MSCs from permanent and deciduous human teeth. MSCs are comprised of heterogeneous population thus it is important to characterize them for the presence of stem cell sub-populations. Therefore, we plan to characterize these cells using classical MSCs markers and their regeneration potential *in-vitro*. We believe, the major benefits from the protocol that it could provide a reliable source of stem cells to be used in regeneration of damaged or diseased tissue or organs, generation of patient specific stem cells and iPSCs banking.

### Author`s Contribution

**SN** contributed in sample collection and transportation to research lab, designed and performed the experiments, literature search, statistical analysis, manuscript writing and editing of manuscript.

**FRK** conceived the idea, designed study, obtained specimens, wrote manuscript and reviewed the manuscript.

**RRZ** critically reviewed the manuscript.

**SSL** carried out ordering, conducted pilot experiment, literature search.

**MSK** carried out ordering, conducted pilot experiment, and literature search.

**NM** participated in study design, interpreted data and reviewed manuscript.

**TA** supervised the project, wrote manuscript and critically reviewed the manuscript.

All authors have approved the final version of the manuscript and disclose no conflict of interest.
